# Cellular and molecular mechanisms involved in the neuroprotective effects of VEGF on motoneurons

**DOI:** 10.3389/fncel.2013.00181

**Published:** 2013-10-21

**Authors:** Jerònia Lladó, Laia Tolosa, Gabriel Olmos

**Affiliations:** ^1^Grup de Neurobiologia Celular, Departament de Biologia, Universitat de les Illes BalearsPalma de Mallorca, Spain; ^2^Institut Universitari d'Investigacions en Ciències de la Salut (IUNICS), Universitat de les Illes BalearsPalma de Mallorca, Spain

**Keywords:** VEGF, motoneuron, ALS, AMPA receptors, excitotoxicity, Akt

## Abstract

Vascular endothelial growth factor (VEGF), originally described as a factor with a regulatory role in vascular growth and development, it is also known for its direct effects on neuronal cells. The discovery in the past decade that transgenic mice expressing reduced levels of VEGF developed late-onset motoneuron pathology, reminiscent of amyotrophic lateral sclerosis (ALS), opened a new field of research on this disease. VEGF has been shown to protect motoneurons from excitotoxic death, which is a relevant mechanism involved in motoneuron degeneration in ALS. Thus, VEGF delays motoneuron degeneration and increases survival in animal models of ALS. VEGF exerts its anti-excitotoxic effects on motoneurons through molecular mechanisms involving the VEGF receptor-2 resulting in the activation of the PI3-K/Akt signaling pathway, upregulation of GluR2 subunit of AMPA receptors, inhibition of p38MAPK, and induction of the anti-apoptotic molecule Bcl-2. In addition, VEGF acts on astrocytes to reduce astroglial activation and to induce the release of growth factors. The potential use of VEGF as a therapeutic tool in ALS is counteracted by its vascular effects and by its short effective time frame. More studies are needed to assess the optimal isoform, route of administration, and time frame for using VEGF in the treatment of ALS.

## INTRODUCTION

Vascular endothelial growth factor (VEGF) was originally described as a factor with a regulatory role in vascular growth and development (reviewed by Carmeliet, 2003; Ferrara, 2004); currently, it is also known for its direct effects on a variety of neuronal cells, modulating neuronal migration, neuritic outgrowth, axon guidance and neuronal survival (reviewed by Ruiz de Almodovar et al., 2009; Mackenzie and Ruhrberg, 2012; Rosenstein et al., 2012).

The VEGFs form a family of growth factors that includes VEGF-A, VEGF-B, VEGF-C, VEGF-D, VEGF-E, and placental growth factor (Takahashi and Shibuya, 2005). The biological activity of the VEGF family is mediated through the binding to two classes of receptors. The tyrosine kinase receptors include the VEGF receptor-1 (VEGFR1, Flt-1), VEGF receptor-2 (VEGFR2, KDR, Flk-1), and VEGF receptor-3 (VEGFR3, Flt-4). The second class, the non-tyrosine kinase receptors, are the neuropilin-1 (NP-1) and neuropilin-2 (NP-2), which are also receptors for semaphorins, and function as co-receptors for the VEGFRs (reviewed by Carmeliet and Ruiz de Almodovar, 2013).

Vascular endothelial growth factor-A (hereafter referred as VEGF) is expressed in different isoforms in humans, which differ in molecular mass, solubility, receptor affinity, and most likely, in its biological function. VEGF_165_ is the predominant isoform and is secreted as a 45-kDa covalently linked homodimer (reviewed by Bogaert et al., 2006). VEGF is widely expressed throughout the central nervous system. Its expression has been reported in neurons (Ogunshola et al., 2002; Schiera et al., 2007), astroglia (Ijichi et al., 1995), and microglia (Bartholdi et al., 1997). VEGF expression is low in the normal adult spinal cord (Fu et al., 2005); however, it increases in response to injury (Choi et al., 2007). VEGF binds to VEGFR1, VEGFR2, NP-1, and NP-2. VEGFR2 is expressed in many populations of neurons and some glial cells; whereas VEGFR1 is predominantly expressed by activated astrocytes and microglia following acute injury (Ogunshola et al., 2002; Choi et al., 2007; Krum et al., 2008; Ruiz de Almodovar et al., 2009). In addition, direct effects of VEGF on Schwann cells have been described (Sondell et al., 1999). NP-1 and NP-2 are expressed in different types of neurons (Kolodkin et al., 1997; Giger et al., 1998), and also in spinal cord motoneurons (Oosthuyse et al., 2001).

Vascular endothelial growth factor has pro-survival effects on some neuronal cells, protects against experimentally induced cell death (Jin et al., 2000), stimulates axonal growth, and guidance (Sondell et al., 2000; Erskine et al., 2011; Ruiz de Almodovar et al., 2011), stimulates neurogenesis (Jin et al., 2002), regulates neuronal migration (Schwarz et al., 2004; Ruiz de Almodovar et al., 2010), and promotes dendrite patterning and synaptic plasticity (Licht et al., 2010, 2011). In addition to the vascular effects of VEGF protecting motoneurons by ensuring optimal blood supply to brain and spinal cord, it functions as a neurotrophic factor for motoneurons (Oosthuyse et al., 2001; Van Den Bosch et al., 2004). VEGF protects motoneurons from insults such as oxidative stress (Li et al., 2003), hypoxia/hypoglycemia (Van Den Bosch et al., 2004), and glutamate-excitotoxicity (Tovar-Y-Romo et al., 2007; Tolosa et al., 2008; Tovar-Y-Romo and Tapia, 2010).

## ROLE OF VEGF IN AMYOTROPHIC LATERAL SCLEROSIS PATHOGENESIS

The discovery in the past decade that transgenic mice with a homozygous deletion in the hypoxia response element site in the VEGF promoter (VEGF^δ^^/^^δ^ mice) expressed reduced levels of VEGF (25–40% less) and developed late-onset motoneuron pathology reminiscent of amyotrophic lateral sclerosis (ALS), opened a new field of research on this dramatic disease. Interestingly, all the classic features of ALS including misaccumulation of neurofilaments in brainstem and spinal cord motoneurons, degeneration of motor axons, and denervation-induced muscle atrophy can be observed in these mice (Oosthuyse et al., 2001). As expected, mice engineered to overexpress VEGF had a delayed motoneuron degeneration and an increased survival when crossed to the superoxide dismutase-1 (SOD1) mouse model of ALS (Wang et al., 2007). In addition, the reduction in the levels of VEGF in the SOD1 mutant mice by crossbreeding the SOD1 mouse model of ALS with VEGF^δ^^/^^δ^ mice worsened the disease, resulting in a decrease in survival due to more severe motoneuron degeneration and earlier onset of muscle weakness (Lambrechts et al., 2003). Interestingly, in the SOD1 mutant mice model of ALS, mutant SOD1 can disrupt the post-transcriptional regulation of VEGF, leading to decreased production of this neurotrophic factor. This effect seems to be restricted to spinal cord, and the decline in VEGF mRNA levels is apparent before onset of weakness, and is more pronounced at middle and end-stages of the disease (Lu et al., 2007). Together, these results suggest a clear relationship between VEGF expression and the familial forms of ALS linked to SOD1 mutations. It still remains unknown the role that VEGF could play in sporadic ALS. In this sense, genetic studies in humans have indicated that VEGF is a modifier of motoneuron degeneration, as a low-VEGF genotype was associated to an increased susceptibility to ALS (Lambrechts et al., 2009).

It is accepted that the major mediator of the trophic effects on spinal cord motoneurons is VEGFR2 (Tolosa et al., 2008; Tovar-Y-Romo and Tapia, 2010), and the concurrent expression of VEGF and VEGFR2 may suggest autocrine/paracrine effects on these cells (Oosthuyse et al., 2001; Ogunshola et al., 2002; Brockington et al., 2006). Interestingly, both VEGF and VEGFR2 expression is reduced in motoneurons and spinal cord of ALS patients (Brockington et al., 2006). Furthermore, the importance of VEGFR2 has been reinforced by experiments showing increased survival of SOD1 mutant mice after overexpression of VEGFR2 (Storkebaum et al., 2005). These findings support the hypothesis that reduced VEGF signaling may play a role in the pathogenesis of ALS (reviewed by Sathasivam, 2008).

Excitotoxicity is a fundamental mechanism involved in motoneuron degeneration in ALS (reviewed by Van Den Bosch et al., 2006). Defective glutamate transport, causing an abnormally increased extracellular concentration of glutamate and over activation of glutamate receptors, has been proposed as an important mechanism in the excitotoxic process in ALS (Rothstein, 2009). In this regard, a decreased expression of the GLT-1 astroglial transporter has been found in the SOD1 animal models around spinal cord motoneurons (Bendotti et al., 2001; Howland et al., 2002). Excessive calcium influx through α-amino-3-hydroxy-5-methyl-4-isoxazole propionic acid (AMPA) glutamate receptors is the final effector of motoneuron death in the excitotoxic process. Motoneurons are especially vulnerable to AMPA receptor-mediated excitotoxicity both *in vitro* and *in vivo* as they express a high number of Ca^2^^+^-permeable AMPA receptors (Carriedo et al., 1996; Van Den Bosch et al., 2000). The permeability of the AMPA receptor depends upon the GluR2 subunit, which regulates the permeability to calcium: only AMPA receptors lacking GluR2 are permeable to calcium. In this regard, motoneurons express low levels of GluR2 and this renders them vulnerable to AMPA receptor-mediated excitotoxicity (Van Damme et al., 2002). Thus, selective loss of motoneurons can be induced experimentally by intrathecal or intraspinal administration of AMPA receptor agonists (Corona and Tapia, 2004; Sun et al., 2006).

In our laboratory, we used spinal cord organotypic cultures to create a model of chronic glutamate excitotoxicity in which glutamate transporters were inhibited by threohydroxyaspartate (THA) to induce motoneuron death. The exposure of these cultures to THA in the presence of VEGF significantly increased motoneuron survival (Tolosa et al., 2008). Similar results were previously obtained *in vivo* after AMPA-induced chronic excitotoxicity in rat spinal cord (Tovar-Y-Romo et al., 2007). Thus, VEGF protects motoneurons from excitotoxic death; however, it has been recently demonstrated *in vivo* that the therapeutic potential of VEGF against excitotoxicity has a short effective time frame, i.e., VEGF was effective only when administered before the onset of motor symptoms (Tovar-y-Romo and Tapia, 2012).

## MECHANISMS OF VEGF PROTECTION AGAINST EXCITOTOXICITY IN ALS

Matsuzaki et al. (2001) initially identified VEGFR2 as the receptor responsible for the neuroprotective effects of VEGF against excitotoxicity in hippocampal neurons. VEGFR2 is expressed by motoneurons in humans (Brockington et al., 2006), mouse (Oosthuyse et al., 2001), and neonatal (Tolosa et al., 2008) and adult rats (Tovar-Y-Romo and Tapia, 2010), and the anti-excitotoxic effects of VEGF in these cells have also been attributed to this receptor (Bogaert et al., 2006; Tolosa et al., 2008; Tovar-Y-Romo and Tapia, 2010).

The signal transduction pathways activated by VEGF are well-characterized in endothelial cells; however, the knowledge of the signaling pathways involved in the anti-excitotoxic effects of VEGF is still incomplete. Upon ligand binding, VEGFR2 undergoes phosphorylation (Meyer et al., 1999), activating intracellular signaling pathways including phosphatidylinositol 3-kinase (PI3-K)/Akt and mitogen-activated protein kinase/extracellular signal-regulated kinase (MEK)/extracellular signal-regulated kinase (ERK). The relevance of the PI3-K/Akt pathway in the neuroprotective effects of VEGF was first proven on the motoneuron-like NSC34 cell line (Li et al., 2003) and also in SOD1 mutant rats where it was shown to counteract the loss of Akt activity preceding motoneuron degeneration (Dewil et al., 2007b). We demonstrated for the first time in spinal cord organotypic cultures that inhibition of the PI3-K/Akt pathway abolishes the anti-excitotoxic effects of VEGF on motoneurons exposed to a glutamate transporter inhibitor (Tolosa et al., 2008). These results were further confirmed *in vivo* in rats exposed to AMPA (Tovar-Y-Romo and Tapia, 2010). These studies also suggested that the MEK/ERK was less relevant than the PI3-K/Akt signaling pathway, as MEK inhibition had a limited effect on the VEGF-mediated neuroprotection against AMPA-induced excitotoxicity (Tovar-Y-Romo and Tapia, 2010).

Activation of PI3-K by VEGF has additional neuroprotective implications as Akt phosphorylates and activates the cyclic AMP-response element binding protein (CREB), involved in the transcription of the Bcl-2 gene (Pugazhenthi et al., 2000). We demonstrated that excitotoxic conditions are associated to a decreased expression of Bcl-2 in spinal cord cultures, and that VEGF-induced neuroprotection in motoneurons could be related to the restoration, via PI3-K, of Bcl-2 levels in these cultures, and specifically in motoneurons (Tolosa et al., 2008). Bcl-2, besides its ability to block cytochrome *c* release, has been shown to increase calcium uptake and buffering capacity in mitochondria (Zhong et al., 1993), thus protecting against excitotoxicity. Additionally, it has been shown that Bcl-2 overexpression attenuates motoneuron degeneration in the SOD1 animal model (Azzouz et al., 2000).

Interestingly, it has been suggested that the PI3-K/Akt signaling pathway could be involved in GluR2 subunit assembly into AMPA receptors (Rainey-Smith et al., 2010). In this sense, VEGF has been shown, both *in vitro* and *in vivo*, to increase the expression of GluR2 subunit, thus reducing the permeability of AMPA receptors to calcium, and minimizing the vulnerability of motoneurons to AMPA-mediated excitotoxicity (Bogaert et al., 2010). Thus, a potential mechanism for VEGF protection against excitotoxicity would be through a PI3-K/Akt-mediated insertion of the GluR2 subunit of the AMPA receptor in motoneurons. Astrocytes are able to protect against excitotoxicity by inducing GluR2 expression in motoneurons. Interestingly, mutant SOD1 abolishes the ability of astrocytes to regulate GluR2 and thus, increase the susceptibility of motoneurons to excitotoxicity (Van Damme et al., 2007). It remains unknown if the VEGF-induced insertion of GluR2 could be astrocyte-mediated.

p38 mitogen-activated protein kinase (p38MAPK) belongs to a family of protein kinases activated by a range of stimuli including proinflammatory cytokines and oxidative stress (Mielke and Herdegen, 2000). As increased phosphorylation of p38MAPK has been reported in the spinal cord of SOD1 mutant mice, in motoneurons and glial cells, this kinase has been suggested to play a role in the pathogenesis of ALS (Tortarolo et al., 2003; Bendotti et al., 2004). In addition, a motoneuron specific death pathway, involving Fas, p38MAPK, and neuronal nitric oxide synthase activation has been described. Motoneurons from SOD1 mutant mice displayed increased susceptibility to activation of this pathway (Raoul et al., 2002).

Rho-mediated calcium-dependent activation of p38αMAPK has been described as a trigger of excitotoxic cell-death (Semenova et al., 2007). In this regard, it has been shown that VEGF is able to block the AMPA-induced phosphorylation of p38MAPK (Tovar-Y-Romo and Tapia, 2010), thus identifying another molecular mechanism for the anti-excitotoxic effects of VEGF. However, the sole inhibition of p38MAPK activity is not sufficient to protect motoneurons against excitotoxicity as the anti-excitotoxic effects of VEGF are also dependent on the activation of the PI3-K/Akt pathway (Tovar-Y-Romo and Tapia, 2010). In this regard, PI3-K/Akt has been reported to inhibit the phosphorylation of p38MAPK in an apoptosis signal-regulating kinase 1 (ASK1)-dependent manner (Ichijo et al., 1997; Kim et al., 2001). In agreement with that, our group has demonstrated that VEGF protects motoneurons from serum deprivation-induced cell death through PI3-K-mediated inhibition of p38MAPK phosphorylation (Tolosa et al., 2009). Moreover, the inhibition by VEGF of p38MAPK might protect motoneurons in ALS tissue exerting a dual role both through an indirect effect on glial cells (Tortarolo et al., 2003), and a direct anti-apoptotic effect on motoneurons (Dewil et al., 2007a).

## ROLE OF NON-NEURONAL CELLS IN THE NEUROPROTECTIVE EFFECTS OF VEGF

Astroglia (Oosthuyse et al., 2001) and microglia (Bartholdi et al., 1997) are sources of VEGF in the spinal cord and a role for non-neuronal cells has been described in the onset and progression of the pathology in ALS (Clement et al., 2003; Barbeito et al., 2004; Sargsyan et al., 2005). It has been hypothesized that VEGF may also affect motoneurons through an indirect effect on glial cells, as both astrocytes (Krum et al., 2002) and microglia (Ryu et al., 2009) respond to VEGF stimulation. On the one hand, VEGF may affect the glial release of trophic factors, and thus, indirectly, protect motoneurons (reviewed by Bogaert et al., 2006). On the other hand, VEGF decreases the astroglial activation observed in the SOD1 mouse model of ALS, and also enhances neuromuscular junction formation (Zheng et al., 2007). Moreover, the neuroprotective effects observed with lithium in animal models of ALS could be due, in part, to an upregulation of VEGF in non-neuronal cells, as an increase in VEGF has been observed after lithium exposure in brain astrocytes and endothelial cells (Guo et al., 2009). In spite of these potential neuroprotective effects of VEGF involving non-neuronal cells, recently, it has been demonstrated that, under inflammatory conditions, astrocytic expression of VEGF is a key driver of blood–brain barrier disruption, leading to edema, excitotoxicity, and entry of inflammatory cells (Argaw et al., 2012).

Several *in vivo* and *in vitro* studies have indicated that VEGF induces adult neurogenesis (Jin et al., 2002; Cao et al., 2004). It still remains unknown if VEGF *in vivo* induces neurogenesis directly in neural stem cells or indirectly through effects on endothelial cells or other cell types (reviewed by Carmeliet and Ruiz de Almodovar, 2013). The potential of VEGF generating new neurons, together with its ability to induce axon growth could be relevant in its neuroprotective effects on ALS.

## POTENTIAL USE OF VEGF AS A THERAPEUTIC TOOL IN ALS

Vascular endothelial growth factor clearly ameliorates the illness in the mutant SOD1 mice and rats (Azzouz et al., 2004; Storkebaum et al., 2005; Wang et al., 2007), supporting the hypothesis of a role for VEGF in ALS. VEGF has been administered to animals using different strategies. VEGF was administered using lentiviral vectors (intramuscularly delivered and then retrogradely transported) increasing the life expectancy of ALS mice. The treatment was more effective when initiated before disease onset (Azzouz et al., 2004).

Intravenous administration of VEGF induces vascular effects: blood vessel growth or blood–brain barrier alterations (Young et al., 2004). To avoid these problematic side-effects, continuous intracerebroventricular (i.c.v.) administration of VEGF in ALS rats was performed. VEGF at doses between 0.2 and 2 μg·kg^-^^1^·day was safe as it did not induce angiogenesis or inflammation. Besides, it was demonstrated that VEGF diffused from the cerebrospinal fluid to the spinal cord parenchyma, reaching motoneurons, and thus, improving motor performance and prolonging survival of SOD1 rats (Storkebaum et al., 2005). Thus, either retrograde (Azzouz et al., 2004) or paracrine (Storkebaum et al., 2005) delivery of VEGF is effective in the animal models of ALS.

Poesen et al. (2008) have demonstrated that the VEGF-B_186_ isoform is also expressed in the nervous system, has less vascular effects, and also functions as a neuroprotective factor for motoneurons. Interestingly, in contrast to VEGF-A, the presence of VEGF-B is not critical for survival or for motoneuron development in physiological conditions; however, crossing VEGF-B^-^^/^^-^ mice with SOD1 mice aggravated motoneuron degeneration. The effect of VEGF-B_186_ seems to be mediated by VEGFR-1, which is also expressed by spinal cord motoneurons, indicating that they can respond to this VEGF-B isoform. In addition, as VEGFR1 is also expressed on astrocytes, an indirect effect on glia could not be ruled out. Finally, the authors demonstrated that i.c.v. delivery of VEGF-B ameliorated the disease in SOD1 rats without exhibiting side vascular effects (Poesen et al., 2008).

Taking advantage of these previous studies on animal models of ALS, ongoing clinical trials are essaying direct i.c.v. administration of VEGF in humans. Clinical trials on phase I/II investigate safety parameters in ALS patients and those on phase II/III are intended to evaluate the efficacy to increase lifespan ().

## CONCLUSION

**FIGURE 1:**
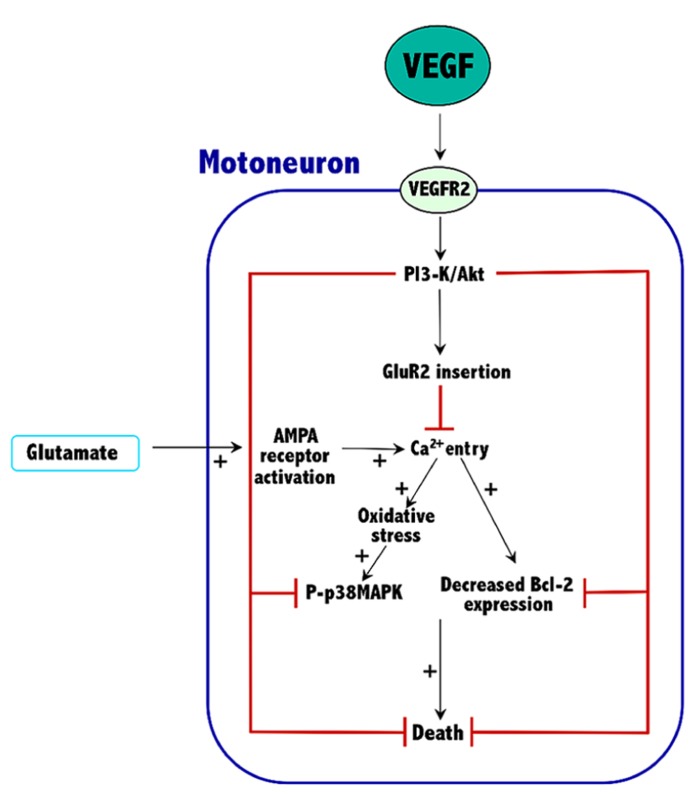
**Mechanisms involved in the neuroprotective effects of VEGF against excitotoxicity in spinal cord motoneurons.** The plus symbols (+) indicate the signaling pathways that are potentiated by excitotoxicity. See Section “Conclusion” for further details.

Current knowledge indicates that VEGF can prevent excitotoxic motoneuron death, thus prolonging survival in an animal model of ALS. These effects are VEGFR2-mediated and involve the activation of the PI3-K/Akt signaling pathway, which results in an increased expression of both Bcl-2 and the GluR2 subunit of AMPA receptors. The overall effect of these proteins would be to reduce the excessive entry of calcium characteristic of the excitotoxic process. Thus, Bcl-2 increases the calcium uptake and the buffering capacity of mitochondria, and GluR2 assembly into AMPA receptors reduces their permeability to calcium. By reducing calcium levels into motoneurons of ALS tissue, VEGF reduces oxidative stress and p38MAPK activity, thus improving survival (**Figure 1**).

Although many of the experimental evidences of the benefits of VEGF in ALS are taken from *in vitro* or *ex vivo* experiments, the promising results obtained in animal models of familial ALS substantiate a potential use of VEGF as a therapeutic tool. However, its effectiveness may be counteracted by its vascular effects and by its expected short effective time frame (Tovar-y-Romo and Tapia, 2012). Clearly, more studies are needed to assess the optimal family member/isoform, the route of administration and the time frame for using VEGF in the treatment of ALS. In addition, a better understanding of the cellular and molecular mechanisms involved in the neuroprotective effects of VEGF will be crucial for its therapeutic development.

## Conflict of Interest Statement

The authors declare that the research was conducted in the absence of any commercial or financial relationships that could be construed as a potential conflict of interest.
